# Gleanings in Science and Medicine

**Published:** 1893-05-13

**Authors:** 


### Gleanings in Science and Medicine.

New York is being much exercised as to which, after all, is the better mode of rapid transit, the "elevated" or the "underground" roads. This points to the fact that the former have not proved themselves quite the perfect acquisition to the city which, prior to erection, they were declared to be. The noise from this overhead traffic is now found to be a serious drawback, as is the obstruction of light and air to the streets below, combined with the accumulation of dust and dirt and cinders which fly about.

The Dean of the Medical Faculty of Hamburg has published the following paragraph, in explanation of certain questions raised by the town, as to the remuneration for the medical students who served in the Hamburg cholera epidemic. "The Cholera Commission of the Senate of Hamburg, in a letter addressed to the undersigned Dean, expresses its thanks to all the students of medicine who assisted in combating the cholera epidemic, and at the same time intimates the wish to send the undermentioned fee to all those who worked in the Hamburg hospitals in the belief that their services were to be remunerated at the rate of 20 marks a day."

Monsieur Jules Simon asserts that "the" secret *par excellence* which conduces to long life is mental employment —"intellectual work," in other words. If, indeed, this is the only condition which is required, M. Simon has put longevity into the power of us all. It is not so very long ago, however, that we were told with equal certainty that if we eat lemons we should not die; now we are told that if we are intellectual we shall live. The love of life is strong in us, and the secret of reaching to a ripe old age is ensured by pursuing either of those courses—so say the originators of the receipts. Monsieur Simon has much to substantiate his theory, but the elixir of life is undiscovered yet. In pointing to the hale and hearty octogenarians around him in the Paris Institute, as in the Academy of Moral Sciences, M. Simon forgets the names of the illustrious dead, whose memories are immortalised in our minds, but whose brains did not triumph over matter, and never lived to mature to a long perfection what earlier life gave promise of. William Shakespeare, Raphaelle, Shelley, Mozart, and Schiller are amongst the number who cannot be included amongst those whose brain work earned long lives, though Monsieur Simon would answer perhaps that they are only the exceptions which go to prove the rule.

The new "train de luxe" of the German Emperor is a marvel of comfort and elegance. It is a palace on wheels, and in his Imperial Majesty's future journeys few of the luxuries of his ordinary stationary life will be lost on the metal rails. The drawing-room library is hung with Gobeline tapestry, and the dining-room is panelled with oak. Each sleeping-room has its bath, whilst a kitchen fitted with every modern appliance, and a reception-room decorated with marble statuary, is included in the twelve carriages completing this fin de siècle train, which cost the very excusable sum of £90,000.

The English were the first people to take up the cause of dumb animals. In future the Society for Prevention of Cruelty to Animals is to have a Royal President in the Duke of York. But the Italians have also had their sense awakened as to what is expected of all highly civilised nations. For many years the cruelty of the Neapolitans to their animals, to their horses especially, has been a by-word against them. Their horses have been overworked and neglected, ill-fed, ill-housed. Attention from an outside source having been called to this distressing state of things, a society for the suppression of cruelty to animals was started. The Princess Mele Barese of Casa Mele presiding. This society in its yearly report gives a good account of work done by its members, and we may soon hope for a happier condition of dumb beasts in Italy.

The sanitary arrangements of German schools have not been above criticism. English parents know this to their cost, for how many physical wrecks have been returned to them in lieu of the rosy-cheeked children whom they committed to the keeping of schools in that country? This century has recognised the necessity of a conversance with German, but how many of us have acquired the language at the cost of our health? The intellectual gain has been often strangely paid for physically. Hygiene has been the last consideration of these establishments. The food supplied, and the drainage (or rather, the want of both), have not been in keeping with a very high intellectual standard. We are glad to hear that at last this state of things has received a local recognition, and an excellent resolution has just been passed by the Teachers' Association at Berlin, that all schools in the German capital are to receive careful sanitary investigation at the hands of medical men, public officials, and engineers.

